# Advances in the study of macrophage polarization in inflammatory immune skin diseases

**DOI:** 10.1186/s12950-023-00360-z

**Published:** 2023-10-12

**Authors:** Tingting Xia, Shengping Fu, Ruilin Yang, Kang Yang, Wei Lei, Ying Yang, Qian Zhang, Yujie Zhao, Jiang Yu, Limei Yu, Tao Zhang

**Affiliations:** 1 Key Laboratory of Cell Engineering of Guizhou Province, Affiliated Hospital of Zunyi Medical University, Zunyi, Guizhou China; 2 Department of Dermatology, Affiliated Hospital of Zunyi Medical University, Zunyi, Guizhou China; 3 Department of Human Anatomy, Zunyi Medical University, Zunyi, Guizhou China; 4 Department of Laboratory Medicine, Affiliated Hospital of Zunyi Medical University, Zunyi, Guizhou China

**Keywords:** Macrophage polarization, Signaling pathway, Inflammation, Immunity, Skin diseases

## Abstract

When exposed to various microenvironmental stimuli, macrophages are highly plastic and primarily polarized into the pro-inflammatory M1-type and the anti-inflammatory M2-type, both of which perform almost entirely opposing functions. Due to this characteristic, macrophages perform different functions at different stages of immunity and inflammation. Inflammatory immune skin diseases usually show an imbalance in the M1/M2 macrophage ratio, and altering the macrophage polarization phenotype can either make the symptoms worse or better. Therefore, this review presents the mechanisms of macrophage polarization, inflammation-related signaling pathways (JAK/STAT, NF-κB, and PI3K/Akt), and the role of both in inflammatory immune skin diseases (psoriasis, AD, SLE, BD, etc.) to provide new directions for basic and clinical research of related diseases.

## Introduction

The skin, the largest and most exposed organ of the body, must react appropriately to environmental stimuli through a variety of mechanisms to create an immunological and mechanical barrier to the environment. Skin inflammation is the body’s response to different types of injuries, including infections. It involves a complex immune response that includes processes such as recruiting immune cells, responding to changes in blood vessels, and releasing inflammatory factors that aid in fighting pathogens, repairing damaged tissues, and maintaining balance within the body [1]. However, if the immune response becomes too intense, it can lead to chronic inflammation and autoimmune skin diseases. Macrophages are key players in the inflammatory immune response of the skin, and they have a vital role in maintaining the body’s balance while also serving as the primary defense against foreign microorganisms and pathogens. Macrophages regulate skin homeostasis by responding to stimulation and creating polarization through various signaling pathways, which support both pro- and anti-inflammatory responses. In recent years, numerous research studies have identified a significant connection between the polarization of macrophages and the emergence and advancement of various inflammatory immune disorders. Consequently, this paper aims to compile information on macrophage polarization, associated signaling pathways, and their impact on inflammatory immune skin disease, hoping to offer valuable references for both fundamental research and clinical treatment of macrophage polarization-related skin diseases.

## Origin, polarization types, and functions of macrophages

In response to danger signals, the immune system activates a protective inflammatory immune response that includes various processes such as destroying pathogens, removing cellular debris, repairing tissue, and maintaining body homeostasis [2]. As the body’s first line of defense against diseases, macrophages are crucial because they phagocytose foreign microorganisms and regulate the immune system’s reaction to different pathogens by processing and presenting antigens [3]. Macrophages are derived from circulating monocytes, which originate from hematopoietic stem cells in the bone marrow [4, 5]. It was previously believed that tissue-resident macrophages in both healthy and diseased areas come from circulating monocytes [6]. However, recent studies have shown that most tissue-resident macrophages actually originate from the yolk sac and fetal liver during embryonic development [7]. Macrophage populations differ greatly between tissues and perform important physiological functions specific to their residing tissues. Examples include microglia in the central nervous system, osteoclasts in the bone, Kupffer cells in the liver, alveolar macrophages in the lung, histiocytes in the spleen and connective tissue, tissue macrophages in the intestine, and Langerhans cells in the skin [8]. In general, resident macrophages maintain tissue homeostasis, while macrophages derived from monocytes primarily assist in host defense and pathological signaling [9].

**Fig. 1:**
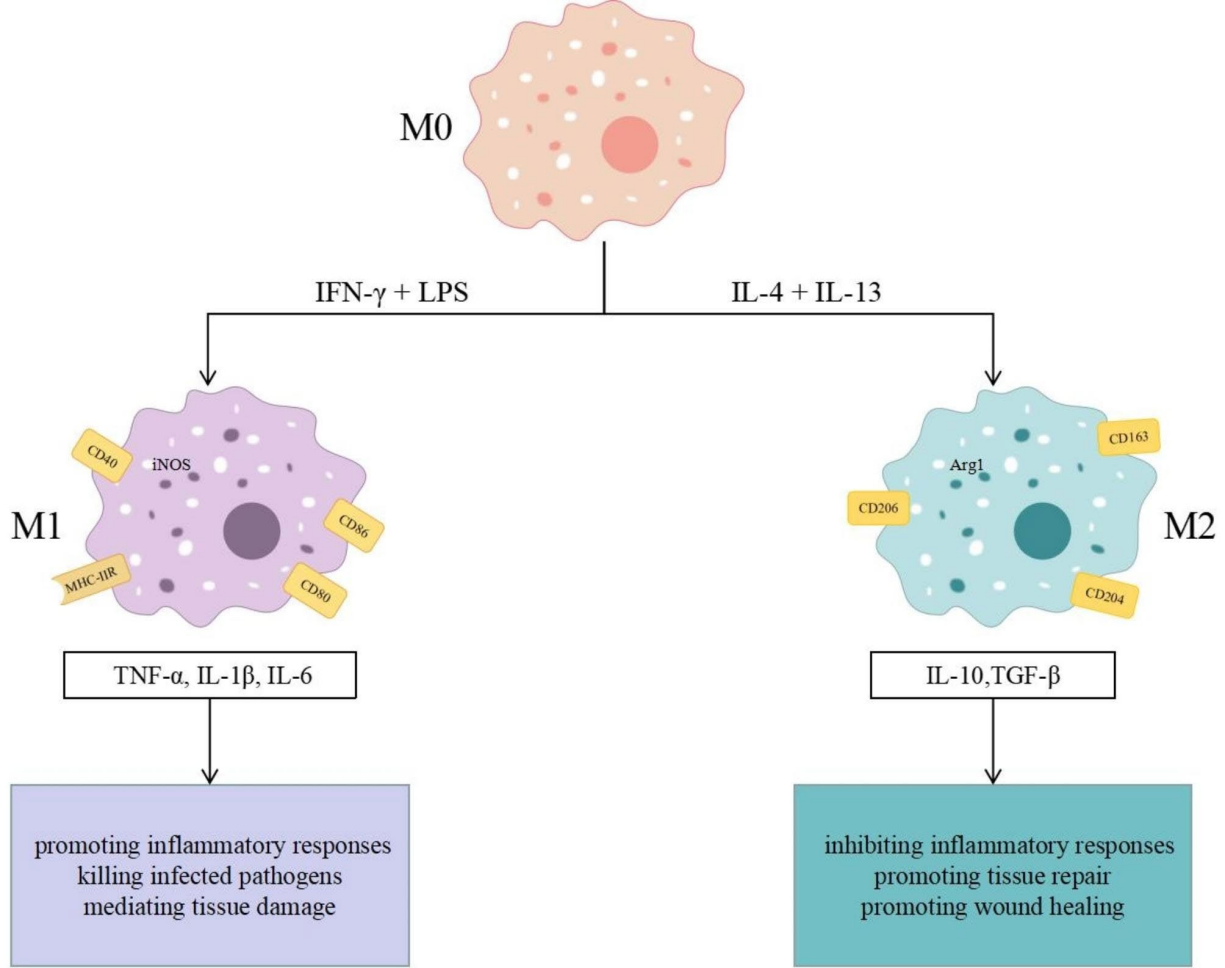
M1-type and M2-type macrophage polarization. Under the influence of IFN-γ and LPS, M0 macrophages polarize into M1-type, leading to the activation of an inflammatory response, elimination of pathogens, and the initiation of tissue damage through the release of pro-inflammatory molecules like TNF-α, IL-1β, and IL-6. Conversely, when exposed to IL-4 and IL-13, M0 macrophages shift towards M2-type, resulting in the suppression of inflammation, promotion of tissue repair, and facilitation of wound healing through the secretion of anti-inflammatory molecules such as IL-10 and TGF-β

Macrophages can adjust their phenotype and function in response to changes in their surroundings, which is known as macrophage polarization. This concept has been gaining a better understanding in the field of inflammation research. Depending on the expression of certain surface receptors and the secretion of specific molecules, macrophages can be polarized into two phenotypes, namely classically activated M1-type macrophages (pro-inflammatory) and alternative activated M2-type macrophages (anti-inflammatory) [10]. M1-type macrophages constitute the first line of defense against microorganisms or pathogens. They are mainly found in an inflammatory environment dominated by toll-like receptors (TLR) and interferon (IFN) signaling and are involved in promoting Th1 responses, promoting inflammatory responses in the early stages of inflammation, eliminating intracellularly infected pathogens, causing tissue damage through reactive oxygen species, and adversely affecting tissue regeneration and wound healing [11]. M1-type macrophages are typically induced by the combination of IFN-γ and bacterial lipopolysaccharide (LPS), which leads to the secretion of pro-inflammatory factors, such as tumor necrosis factor (TNF)-α, IL-1β, IL-6, nitric oxide synthase (iNOS), chemokines, and increased expression of certain cell surface markers like CD40, CD80, CD86 and major histocompatibility complex class II receptor (MHC-IIR) [12–14]. Among them, CD80/CD86 are cell surface glycoproteins expressed on various antigen-presenting cells, which induce inflammatory responses by binding to CD28 on naive T cells, enhancing proliferative responses and effector functions of T cells [15]. The CD40 receptor, a member of the TNF receptor superfamily found on different immune cells, stimulates the Th1 response when bound to the specific CD40L ligand expressed on the surface of activated CD4 T cells. This interaction is crucial for initiating and maintaining the inflammatory response. The CD40 receptor, a member of the TNF receptor superfamily found on a variety of immune cells, promotes the Th1 response when bound to the specific ligand CD40L expressed on the surface of activated CD4 T cell [16]. This interaction is crucial for initiating and maintaining the inflammatory response. MHC-IIR is found in macrophages, B cells, and dendritic cells, and its primary function is to present exogenously derived peptide antigens to CD4 T cells, playing an important role in initiating adaptive immune responses [17]. M2-type macrophages, also known as reparative macrophages, are involved in promoting Th2 responses with properties that help inhibit inflammatory responses and promote tissue repair and wound healing [11]. The induction of M2-type macrophages is primarily triggered by IL-4 or IL-13. These macrophages overexpress IL-10, transforming growth factor (TGF)-β, vascular endothelial growth factor, epidermal growth factor, and arginase1 (Arg1), as well as increasing the expression of cell surface markers CD163, CD204, and CD206 [12–14]. CD163, a transmembrane scavenger receptor expressed on the surface of monocytes and macrophages, is involved in the clearance of damaged cells and down-regulation of inflammation by directly stimulating the secretion of anti-inflammatory cytokines and facilitating hemoglobin to macrophages to trigger an anti-inflammatory response [18, 19]. CD204 is another scavenger receptor that participates in various pathophysiological processes such as inflammatory responses, innate immunity, host defense, and cancer in vivo by binding to its ligand [20, 21]. CD206, also known as the mannitol receptor, is a transmembrane protein primarily found in macrophages and dendritic cells that mediates phagocytosis and endocytosis uptake of bacterial, protozoan, fungal, and viral antigens and plays an important role in the regulation of innate immunity, inflammatory responses, and homeostasis in the body [22, 23]. Therefore, the polarization status of macrophages can be distinguished by examining the secretion profile of macrophages and surface molecular markers (Fig. 1).

Macrophages are capable of adapting to different microenvironments and producing various bioactive substances that can either promote or suppress inflammation. During the acute phase of inflammation, macrophages polarize towards M1-type, inducing inflammatory response and the release of pro-inflammatory mediators that aid in the elimination of pathogens and damaged cells. Conversely, in the late phase of inflammation, macrophages transition to the M2 phenotype, producing anti-inflammatory cytokines that reduce the inflammatory response, facilitate tissue regeneration, and restore homeostasis in the body [24]. The polarization of T cells into Th1 or Th2 subsets is closely linked to the M1 or M2 phenotype of macrophages in humans, respectively, and together they work in coordination to maintain homeostasis. Any imbalance in this coordination can result in pathological inflammatory responses and associated diseases [25]. Consequently, modulating the ratio of M1 to M2 macrophages holds promise as a novel therapeutic approach for inflammatory immune disorders.

## Signaling pathways involved in the regulation of inflammatory responses by macrophage polarization

Macrophage polarization is regulated through the activation of several interrelated cellular signaling pathways. The main polarization-related pathways involved in inflammation include janus kinases (JAK)/signal transducer and activator of transcription (STAT) signaling pathway, nuclear factor-κB (NF-κB) signaling pathway, phosphoinositide 3-kinase (PI3K)/protein kinase B (Akt) signaling pathway, etc.

### JAK/STAT signaling pathway

The scientific community now widely accepts that the development of many inflammatory immune diseases is connected to the JAK/STAT pathway [26]. JAK/STAT is a major signaling pathway utilized by more than 70 cytokines and is involved in vital biological processes such as cell proliferation, differentiation, apoptosis, and immune regulation [27]. Inflammatory factors are closely linked to the JAK/STAT signaling pathway. The pathway can be activated by inflammatory factors, and in turn, it can affect the expression of these factors. In this pathway, the STAT protein family is an important class of transcription factors, consisting of seven main members [28], among them, the relevant STAT members of macrophage polarization that regulate the inflammatory response include STAT1, STAT3, and STAT6. When IFN-γ and IL-12 bind to their receptors, JAK is activated, leading to the phosphorylation of STAT1. This promotes the polarization of M1-type macrophages and the production of pro-inflammatory factors [29]. On the other hand, IL-4 and IL-13 increase the expression of STAT6, while IL-6 increases the expression of STAT3. Both STAT6 and STAT3 promote the polarization of M2-type macrophages and the production of anti-inflammatory factors [30, 31]. In summary, M1-type polarization is closely associated with STAT1, whereas M2-type macrophage polarization is influenced by the increased expression of STAT3 and STAT6.

### NF-κB signaling pathway

NF-κB acts as a “master switch” for the expression of various pro-inflammatory molecules. When this pathway is dysregulated, it can contribute to the development of a wide range of autoimmune and inflammatory diseases [32]. Studies have shown that TLRs recognize conserved pathogenic microbial products, known as pathogen-associated molecular patterns, which trigger inflammatory immune responses and host defense mechanisms [33]. TLRs on the surface of macrophages bind to lipopolysaccharides (LPS) and activate the classical NF-κB pathway through either the MyD88-dependent pathway or the interferon regulatory factor (IRF) 3 pathway. As a result, NF-κB p65/p50 enters the nucleus and controls the polarization of M1-type macrophages [34]. This process leads to the transcription of pro-inflammatory factors such as IL-1β, IL-6, and TNF-α, thereby amplifying inflammatory signals [35]. In addition, there is a close relationship between NF-κB and JAK/STAT pathway, with STAT1 shown to activate the transcriptional activity of NF-κB [36] and mutual crosstalk between STAT3 and NF-κB regulating M1/M2 homeostasis [37–39].

### PI3K/Akt signaling pathway

The PI3K/Akt signaling pathway is a crucial pathway that controls inflammatory reactions, regulates macrophage polarization, and is crucial for the inflammatory immune response [40]. In macrophages, stimuli such as growth factors and cytokines can be signaled through this pathway [41, 42]. The inhibition of M1-type macrophage polarization and the promotion of M2-type macrophage polarization were observed to occur upon activation of this signaling pathway [43]. This effect was supported by the finding that this signaling pathway negatively regulated TLR and NF-κB signaling and positively regulated STAT3 signaling in macrophages [44, 45]. However, in accordance with various Akt isoforms, the PI3K/Akt signaling pathway differentially promotes macrophage polarization. The three components of Akt, Akt1, Akt2, and Akt3, are 80% homologous and each performs a different regulatory task [46]. Akt1 deletion promotes macrophage polarization toward M1-type and increases the expression of pro-inflammatory factors iNOS, TNF-α, and IL-6, while Akt2 deletion promotes macrophage polarization toward M2-type and enhances the expression of anti-inflammatory factor IL-10; Akt3 deletion reduces M2-type macrophage infiltration and hinders skin wound healing [47, 48].

**Fig. 2:**
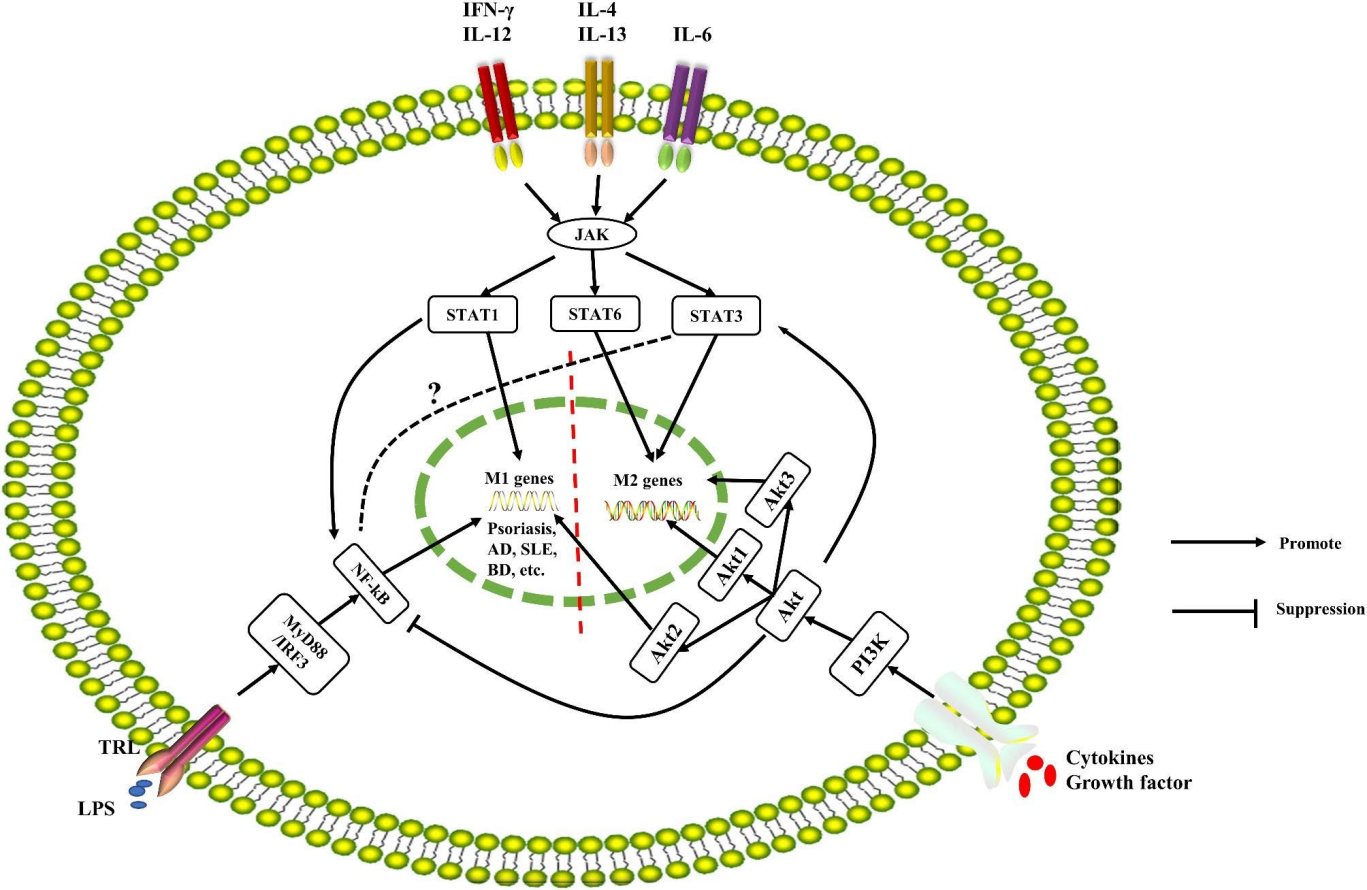
Inflammation-related signaling pathways associated with macrophage polarization. The JAK/STAT1, NF-κB, and PI3K/Akt2 signaling pathways play a role in promoting M1 polarization of macrophages. On the other hand, the JAK/STAT3 or JAK/STAT6 and PI3K/Akt1 or PI3K/Akt3 signaling pathways are involved in promoting M2 polarization of macrophages. These signaling pathways can interact with each other, leading to a combined effect in determining the direction of macrophage polarization

In summary, the JAK/STAT1, NF-κB, and PI3K/Akt2 signaling pathways promote macrophage M1 polarization, increase the production of pro-inflammatory factors IL-1β, IL-6, TNF-α and iNOS, etc., and initiate or exacerbate the inflammatory response; while the JAK/STAT3 or JAK/STAT6 and PI3K/Akt1 or PI3K/Akt3 signaling pathways promote macrophage M2 polarization, increase the production of anti-inflammatory factors such as IL-10 and TGF-β, and suppress the inflammatory response. In turn, these three types of signaling pathways interfere with one another and jointly determine the direction of macrophage polarization (Fig. 2).

## Macrophage polarization regulates inflammatory immune skin diseases

Although inflammation plays a vital role in the body’s defense against external invaders or the control of the immune system’s response, it can also have a harmful effect on the body by impairing normal tissue function and even resulting in organ failure. This results in the emergence of numerous autoimmune and chronic inflammatory diseases. A group of systemic or organ-specific chronic inflammatory skin diseases known as “inflammatory immune skin diseases”, whose pathogenesis is not fully understood, are caused by abnormal inflammatory responses and immune dysregulation [49]. Macrophages are key cells that coordinate chronic inflammation, and macrophage polarization takes place during the onset, development, progression, and remission of inflammatory diseases [50]. According to the studies listed below, macrophage polarization can regulate inflammatory immune skin diseases.

### Psoriasis

With a global prevalence of about 2%, psoriasis is a chronic, relapsing immune inflammatory skin disease also known as the “cancer that never dies“ [51]. Important cytokines like IL-17, IL-22, IL-23, and TNF-α are involved in the pathogenesis of psoriasis, as well as other crucial transduction pathways like the NF-κB and STAT signaling pathways. These pro-inflammatory factors are major drivers of psoriasis pathogenesis through signaling pathways [52, 53]. Kaempferia parviflora is a folk medicine widely used in Southeast Asia for its anti-inflammatory and anti-allergic effects [54]. Inhibiting the LPS-induced NF-κB pathway allows Kaempferia to prevent keratinocytes and macrophages from producing the pro-inflammatory factors TNF-α, IL-1, IL-6, IL-17, IL-22, and IL-23, indicating that this plant may be a promising candidate for the creation of anti-psoriasis medications [55]. This mechanism of reducing inflammation is closely connected to the polarization of macrophages. It is widely accepted that M1-type macrophages produce cytokines that promote inflammation, while M2-type macrophages produce cytokines that have anti-inflammatory properties. The balance between these two types of macrophages plays a crucial role in determining the progression of various inflammatory diseases, including psoriasis [56]. In one study, a circular RNA called hsa_circ_0004287 was found to inhibit the polarization of M1-type macrophages in vitro. Additionally, when a plasmid containing hsa_circ_0004287 was topically applied to mice with psoriasis-like symptoms, it resulted in a reduction of skin inflammation [57]. Traditional Chinese medicine has shown remarkable success in treating psoriasis in recent years, and figuring out how it works is a hot area of research right now. The herbal PSORI-CM02 formulation, which contains Rhizoma Curcumae, Radix Paeoniae rubra, Sarcandra glabra, Rhizoma Smilacis glabrae, and Fructus mume [58] has been shown to significantly improve imiquimod-induced skin lesions in psoriasis-like mice, and this process was accomplished by controlling STAT1 and STAT6 expression to lessen M1-type macrophage infiltration [59]. By preventing M1-type macrophage polarization in psoriasis and lowering the production of psoriasis-related cytokines, purpurin plus methotrexate has also been shown to protect against psoriasis [60]. In addition to typical M1- and M2-type macrophages, Hou et al [61] identified a unique pathogenic macrophage subpopulation driven by IL-23, referred to as M(IL-23)-type macrophages, which highly expressed IL-17 A, IL-22 and IFN-γ. With the help of the STAT3 and retinoid-related orphan receptor-γT pathway, IL-23 induced the expression of IL-17 in macrophages, and the Th1-related key transcription factor T-bet mediated the production of IFN-γ, both of which significantly worsened skin lesions in mice with psoriasis-like symptoms. Based on these studies, it is suggested that treating psoriasis can be accomplished by decreasing the production of inflammatory factors and adjusting NF-κB and STAT signaling pathways to prevent the polarization of M1-type macrophages or infiltration of M(IL-23) cells.

### Atopic dermatitis (AD)

Up to 20% of children and 5% of adults can develop AD, an immune-driven chronic pruritic inflammatory skin disease that is frequently accompanied by a personal or family history of food allergy, allergic rhinitis/conjunctivitis, or allergic asthma [62]. By controlling the immune response in the skin, macrophages play a direct role in the pathogenesis of AD, and atopic mice observed upregulation of M1-type macrophage markers and downregulation of M2-type markers [63]. Hsa_circ_0004287, which is overexpressed specifically in macrophages, suppressed M1-type macrophage polarization, which reduced skin inflammation in AD mice in addition to having an impact on psoriasis pathogenesis [57]. By activating the JAK2/STAT3 signaling pathway and encouraging M2-type macrophage polarization, Viola yedoensis Makin, a traditional Chinese medicine with anti-inflammatory properties [64], can significantly improve skin lesions and decrease levels of inflammatory factors IL-1β, TNF-α, and IL-18 while increasing levels of anti-inflammatory factor IL-10 in AD mice [65]. However, an intriguing study discovered that inhibiting both M1- and M2-type macrophages also alleviated AD symptoms [66]. It is still unclear how the inflammatory immune response in AD works because it is extremely complicated. To lay a more solid foundation for macrophage polarization in clinical translation, more in-depth investigation is required to clarify the mechanisms of the function of various macrophage subtypes in AD.

### Systemic lupus erythematosus (SLE)

SLE is a chronic autoimmune inflammatory disease characterized by the presence of autoantibodies against nuclear antigens, immune complex deposits, and tissue damage in the skin, kidneys, heart, and lungs, and its pathogenesis has been shown to be associated with M1-type macrophage polarization [67]. Despite the fact that SLE has been the subject of extensive research, its pathogenesis is still unknown, and no effective medications have yet to be discovered [68]. According to Labonte et al.‘s research [69], SLE patients’ bone marrow contained more M1-type macrophages that expressed STAT1, SOCS3, and fewer M2-type macrophages that expressed STAT3, STAT6, and CD163. Exosomes derived from human umbilical cord mesenchymal stem cells decreased TNF-α and IL-1β levels, promoted M2-type macrophage polarization, and increased Treg cell production in the spleen in both in vitro and in vivo experiments, thereby improving nephritis and other serious organ damage and achieving the goal of treating SLE [70, 71]. Therefore, the immune inflammatory response to SLE can be improved by inhibiting M1-type macrophages and activating M2-type macrophages. According to one study, successive transplanting of M2-type macrophages into mice significantly lessened the severity of SLE [72]. Azithromycin has also been shown to inhibit the secretion of pro-inflammatory factors IL-1β, IL-6, and TNF-α, promote the production of anti-inflammatory factor IL-10, and stimulate macrophage M2 polarization through the PI3K/Akt signaling pathway, as well as suppress the immune inflammatory response in SLE [73]. Azithromycin has a good safety profile and is frequently used in clinical practice. A fresh approach to treating SLE patients may emerge from further investigation of its pharmacological effects and assessment of its therapeutic efficacy. These studies demonstrated that M1-type macrophages contribute to tissue injury while M2-type macrophages are involved in tissue healing in SLE, suggesting that restoring the balance between M1/M2 macrophages may be a novel therapeutic target for the disease.

### Behcet’s disease (BD)

Recurrent oral/genital ulcers, skin lesions, ocular damage, and other systemic manifestations are key features of BD, which is a chronic, multisystemic, inflammatory immune vasculitis [74]. BD patients have an overactive immune system and multisystem inflammatory damage, which is primarily shown by an increased inflammatory response and overexpression of pro-inflammatory cytokines like TNF-α, IL-1β, IL-6, IL-12, and IL-18 [75]. In addition, impaired secretion of the anti-inflammatory factor IL-10 is also associated with BD pathogenesis [76]. The ability of macrophages to polarize into different phenotypes, which is a result of their plasticity, allows them to play a significant role in both promoting and suppressing inflammatory processes. The M1/M2 macrophage ratio and the M1-type macrophage phenotype were found to be upregulated in BD mice when compared to normal mice in a study of herpes simplex virus-induced BD mice [77]. The inflammatory alterations in BD may be brought on by BD serum factors [78]. A recent study showed that BD serum polarized macrophages toward M1-type by activating the NF-κB signaling pathway, driving Th1 differentiation, and promoting overexpression of IL-12 and TNF-α [79]. This dysregulation of M1/M2 macrophage homeostasis is associated with abnormal expression of the aryl hydrocarbon receptor (AHR) [80], the latter is a ligand-activated transcription factor. According to Palizgir et al.‘s research, patients with BD had lower levels of monocyte-derived macrophages and in vitro-induced M1-type macrophage AHR mRNA expression than individuals without the condition [81]. There is an urgent need to comprehend the pathogenesis of BD and develop new therapeutic targets because the therapeutic effect of BD is still not satisfactory. As a new therapeutic target for BD, macrophage polarization may make significant strides in terms of BD prognosis improvement and disease burden reduction.

### Others

Acne is a globally common chronic inflammatory immune skin disease that can occur at any age [82]. 5-Aminoketovaleric acid photodynamic therapy is clinically effective and safe in the treatment of patients with severe acne, but its exact mechanism is unknown. A very interesting study revealed that this therapy significantly upregulated the expression of various inflammation-related genes, triggered the polarization of macrophages to M1-type both in vitro and in vivo, and exerted its therapeutic effect by enhancing the intense inflammatory response and breaking chromaffin cells [83], providing new insights into the study of inflammatory-immune skin diseases.

Rosacea is another immune-mediated, chronic inflammatory skin disease that primarily affects the center of the face [84]. Although the exact cause of this condition is unknown, M1-type macrophages and their pro-inflammatory effects may play a role [85]. Zhou et al [86] found increased local infiltration of macrophages in rosacea mice and demonstrated that guanylate-binding protein 5 (GBP5) is an important gene controlling macrophage infiltration. They also proved that silencing GBP5 can achieve therapeutic effects by inhibiting M1-type macrophage polarization and the expression of pro-inflammatory factors IL-1, iNOS, and TNF-α through the NF-κB signaling pathway.

Overall, increased M1-type macrophages or increased M1/M2 macrophage ratio can contribute to or exacerbate inflammatory immune skin diseases like psoriasis, AD, SLE, and BD. Thus, it is anticipated that suppressing M1-type macrophages and achieving a balance in the M1/M2 macrophage ratio will prevent or treat these related diseases.

## Summary and Outlook

In skin tissue, macrophages are key players in tissue homeostasis and immune surveillance, mobilizing immune activation in reaction to microbial invasion and supporting wound healing to restore damaged tissue. In terms of pathogenesis, macrophages, as essential mediators and coordinators of chronic inflammation, are crucial mediators in various diseases. The critical mechanism by which they exert these pathophysiological effects is macrophage polarization. Large amounts of pro- or anti-inflammatory cytokines and chemokines are produced by M1- and M2-type macrophages in response to their respective activators, activating numerous related signaling pathways and carrying out their regulatory functions. Existing research has demonstrated that macrophage polarization can contribute to the onset and development of several inflammatory immune skin diseases, such as psoriasis, AD, SLE, BD, etc. The proportion of M1/M2 type macrophages is elevated in these diseases, and M1-type macrophages dominate in the ongoing development and destructive cycle of the inflammatory response. By targeting molecules in signaling pathways like JAK/STAT, NF-κB, PI3K/Akt, and the local microenvironment, macrophages can be converted to the appropriate phenotype to regulate the onset, progression, and outcome of inflammatory diseases. The mechanisms underlying macrophage polarization in these inflammatory skin diseases, however, are still not yet fully elucidated, and therapies that target macrophage polarization are still in their infancy. Therefore, an in-depth study of macrophage polarization and its function in inflammatory immune dermatoses can provide us with a more thorough comprehension of the pathogenesis of associated dermatoses, which can then provide valuable references for the prevention and treatment of such diseases.

## Data Availability

Not applicable.

## Abbreviations

AD: atopic dermatitis
Akt: protein kinase B
Arg1: arginase1
BD: Behcet’s disease
IFN: interferon
IL: interleukin
iNOS: nitric oxide synthase
JAK: janus kinases
LPS: lipopolysaccharide
NF-κB: nuclear factor kappaB
PI3K: phosphoinositide 3-kinase
SLE: systemic lupus erythematosus
STAT: signal transducer and activator of transcription
TGF: transforming growth factor
TLR: toll-like receptor
TNF: tumor necrosis factor
